# Utility of a novel inflammatory marker, GlycA, for assessment of rheumatoid arthritis disease activity and coronary atherosclerosis

**DOI:** 10.1186/s13075-015-0646-x

**Published:** 2015-05-09

**Authors:** Michelle J. Ormseth, Cecilia P. Chung, Annette M. Oeser, Margery A. Connelly, Tuulikki Sokka, Paolo Raggi, Joseph F. Solus, James D. Otvos, C. Michael Stein

**Affiliations:** Department of Medicine, Vanderbilt University Medical Center, 1161 21st Avenue South, T-3113 MCN, Nashville, TN 37212 USA; LabCorp, 2500 Sumner Blvd, Raleigh, NC 27616 USA; Department of Rheumatology, University of Eastern Finland, Jyvaskyla Central Hospital, 40620 Jyvaskyla, Finland; Department of Medicine, University of Alberta, 4A7.050, 8440 – 112 Street, Edmonton, AB T6G 2B7 Canada

## Abstract

**Introduction:**

GlycA is a novel inflammatory biomarker measured using nuclear magnetic resonance (NMR). Its NMR signal primarily represents glycosylated acute phase proteins. GlycA was associated with inflammation and development of cardiovascular disease in initially healthy women. We hypothesized that GlycA is a biomarker of disease activity and is associated with coronary artery atherosclerosis in patients with rheumatoid arthritis (RA).

**Methods:**

We conducted a cross-sectional study of 166 patients with RA and 90 control subjects. GlycA was measured from an NMR signal originating from *N*-acetylglucosamine residues on circulating glycoproteins. The relationship between GlycA and RA disease activity (Disease Activity Score based on 28 joints (DAS28)) and coronary artery calcium score was determined.

**Results:**

GlycA concentrations were higher in patients with RA (median (interquartile range): 398 μmol/L (348 to 473 μmol/L)) than control subjects (344 μmol/L (314 to 403 μmol/L) (*P* < 0.001). In RA, GlycA was strongly correlated with DAS28 based on erythrocyte sedimentation rate (DAS28-ESR) and DAS28 based on C-reactive protein (DAS28-CRP) and their components, including tender and swollen joint counts, global health score, ESR and CRP (all *P* < 0.001). The area under the receiver operating characteristic curve for GlycA’s ability to differentiate between patients with low versus moderate to high disease activity based on DAS28-CRP was 0.75 (95 % confidence interval (CI): 0.68, 0.83). For each quartile increase in GlycA, the odds of having coronary artery calcium increased by 48 % (95 % CI: 4 %, 111 %), independent of age, race and sex (*P* = 0.03).

**Conclusion:**

GlycA is a novel inflammatory marker that may be useful for assessment of disease activity and is associated with coronary artery atherosclerosis in patients with RA.

## Introduction

Rheumatoid arthritis (RA) is a chronic inflammatory autoimmune disorder affecting nearly 1 % of the US population. Tight control of disease activity, including monitoring of acute-phase reactants, is important to reduce joint destruction and disability in RA [1]. Currently, many rheumatologists consider that monitoring an acute-phase reactant is standard of care in RA management [2]. Indeed, measurement of C-reactive protein (CRP) or erythrocyte sedimentation rate (ESR) is incorporated into the Disease Activity Score based on 28 joints (DAS28) [3], the core set of RA disease activity measures proposed by the American College of Rheumatology [4] and the American College of Rheumatology/European League Against Rheumatism RA remission criteria [5].

The markers of inflammation most commonly used to assess RA disease activity—ESR and CRP—have disadvantages. For example, ESR is altered by non-inflammatory conditions such as chronic kidney disease [6], pregnancy, anemia, abnormal red blood cell shape or size, and serum protein concentrations [7]. Because some of these confounding influences are unrelated to RA disease activity, the current treat-to-target recommendations include cautions about the use of ESR for monitoring RA activity [2]. Furthermore, CRP exhibits high variability over time in patients without inflammatory diseases [8–10], potentially making it unreliable for assessment of RA disease activity at one time point. Moreover, both ESR and CRP increase with age [11], female sex [12, 13] and body mass index (BMI) [14–17]. Thus, an alternative marker of inflammation not affected by some of these factors would be useful for assessing RA disease activity.

GlycA is a nuclear magnetic resonance (NMR) signal derived from mobile *N*-acetyl methyl groups, specifically the *N*-acetylglucosamine and *N*-acetylgalactosamine moieties, on the carbohydrate side chains of glycosylated proteins [18–20]. GlycA can be measured in the NMR spectra acquired for the quantification of serum or plasma lipoprotein particle numbers [10]. The majority of circulating glycosylated proteins are acute-phase proteins [18], and the main contributors to the GlycA signal are α_1_-acid glycoprotein, α_1_-antitrypsin, haptoglobin, α_1_-antichymotrypsin and transferrin [10]. Glycosylated immunoglobulin is not a main contributor to the GlycA signal, however [10].

GlycA is emerging as a new marker of inflammation and cardiovascular (CV) risk. GlycA concentrations were associated with known inflammatory markers, such as CRP, interleukin-6 (IL-6) and fibrinogen in 5,537 participants of the Multi-Ethnic Study of Atherosclerosis [10]. Moreover, GlycA was associated with incident CV events. For example, for every 1 standard deviation (SD) increase in baseline GlycA concentration, there was a 34 % increased incidence of CV events within the first 6 years of follow-up, independent of traditional risk factors, in over 27,000 women from the Women’s Health Study [21]. Little is known about GlycA concentrations in RA. We hypothesized that GlycA is a marker of disease activity and CV risk in patients with RA.

## Methods

### Study population

We conducted a cross-sectional study that included 166 patients with RA and 90 control subjects from a cohort of patients extensively characterized for CV risk [22]. Recruitment and study procedures, as well as predictors of coronary atherosclerosis, in this cohort have been described previously [22–30]. All subjects were older than 18 years of age, and patients with RA fulfilled the 1987 American College of Rheumatology classification criteria for RA [31]. The RA and control groups were frequency-matched for age, race and sex, and control subjects did not have RA or other inflammatory disease. Patients with RA were identified from an early RA registry, Vanderbilt University Medical Center or local rheumatologist referral, or advertisement. Control subjects were recruited from patient acquaintances, advertisement or volunteer database. Consecutive eligible patients were enrolled. The study was approved by the Vanderbilt University Medical Center Institutional Review Board, and all subjects gave us their written informed consent to participate.

### Clinical and laboratory data

Clinical information, laboratory measurements and coronary artery calcium scores were obtained as described previously [22]. Disease activity of RA was determined on the basis of the DAS28-ESR and DAS28-CRP [3]. Categories of disease activity based on the DAS28-ESR were defined as follows [32]: remission = DAS28 < 2.6, low = DAS28-ESR between 2.6 and 3.2, moderate = DAS28-ESR between 3.2 and 5.1, and high = DAS28-ESR >5.1. Categories of disease activity based on the DAS28-CRP were defined as follows [32]: remission = DAS28-CRP <2.3, low = DAS28-CRP between 2.3 and 2.7, moderate = DAS28-CRP between 2.7 and 4.1, and high = DAS28-CRP >4.1. Prevalent coronary artery disease was defined as a history of myocardial infarction; coronary procedure such as stenting, balloon angioplasty or coronary artery bypass surgery; or angina. Estimated glomerular filtration rate (eGFR) was calculated using the Modification of Diet in Renal Disease Study formula [33]: eGFR (ml/min/1.73 m^2^) = 175 × (serum creatinine) − 1.154 × (age) − 0.203 × (0.742 if female) × (1.212 if African American).

ESR was measured at the Vanderbilt University Medical Center Clinical Laboratory, and high-sensitivity CRP was measured at the same laboratory or by enzyme-linked immunosorbent assay (ELISA) (EMD Millipore, Billerica MA, USA). Tumor necrosis factor α (TNFα) and IL-6 concentrations were measured by multiplex ELISA (EMD Millipore).

### Larsen score measurement

The Larsen score was calculated in 92 patients with RA based on radiographs of the hands and feet as described previously [34]. The radiographs were obtained a median of 1.9 years (interquartile range (IQR): 1.1 to 2.7) prior to study enrollment and scored by a blinded single investigator (TS). Larsen score was quantified by assessing the damage to 20 joints (wrists, first to fifth metacarpophalangeal joints and second to fifth metatarsophalangeal joints), yielding a total score ranging from 0 to 100 [35, 36].

### Coronary artery calcium score measurement

Coronary artery calcium scores were calculated by performing electron beam computed tomography with an Imatron C-150 scanner (GE Medical Systems/Imatron, South San Francisco, CA, USA), as previously described [22], and quantified in Agatston units [37].

### GlycA measurement

NMR spectra were acquired from ethylenediaminetetraacetic acid plasma samples as previously described for the NMR LipoProfile (lipoprotein particle) test at LipoScience (Raleigh, NC, USA) [26, 38]. The NMR Profiler platform is comprised of a 9.4-T (400-MHz ^1^H frequency) spectrometer (Bruker BioSpin, Billerica, MA, USA) with an integrated fluidic sample delivery system. The GlycA signal was quantified using proprietary deconvolution software that uses a non-negative linear least squares algorithm to fit the experimental signal to individual spectral components, including proteins and lipoproteins as well as signals representing the GlycA NMR resonance [10]. GlycA concentrations were quantified from these spectra without knowledge of any subject characteristics. The intra-assay and inter-assay variabilities for GlycA measurement are 1.9 % and 2.6 %, respectively [10].

### Statistics

On the basis of a fixed sample size of 166 patients with RA and 90 control subjects and the SD of GlycA 57 μmol/L in control subjects, our study had 90 % power to detect a difference of approximately 25 μmol/L GlycA concentration between RA and control subjects. On the basis of the Women’s Health Study, a 1 SD increase in GlycA was independently associated with a 34 % increased rate of CV events within 6 years of follow-up [21]; thus, the study had sufficient power for us to detect a clinically meaningful difference in GlycA.

Descriptive statistics were calculated as medians with IQRs (25th to 75th percentiles) for continuous variables and as frequency and proportions for categorical variables. To compare variables between RA and control subjects, the Wilcoxon rank-sum test was used to compare continuous variables and the Pearson *χ*^2^ test was used to compare categorical variables. Correlation between GlycA concentrations and variables of interest was determined by Spearman correlation.

The ability of GlycA concentrations to differentiate between low and moderate to high disease activity, defined on the basis of the DAS28-ESR and DAS28-CRP values, was examined by calculating the area under the receiver operating characteristic (AUC) curve.

The association between quartiles of GlycA concentrations and presence of coronary artery calcium as well as presence of coronary artery disease was assessed by binary logistic regression. In these models, the presence of coronary artery calcium or a previous diagnosis of coronary artery disease was the dependent variable and GlycA was the independent variable. Separate analyses were performed, adjusting for age, race, and sex and adjusting for components of Framingham Risk Score, which we have termed *traditional CV risk factors* (age, sex, total cholesterol, high-density lipoprotein (HDL) cholesterol, smoking status, systolic blood pressure and anti-hypertensive use) [39], and then additionally for the presence of diabetes.

Data analysis was performed using IBM SPSS Statistics version 22 software (IBM, Armonk, NY, USA). Two-sided *P*-values <0.05 were considered statistically significant.

## Results

### Clinical characteristics

**Fig. 1:**
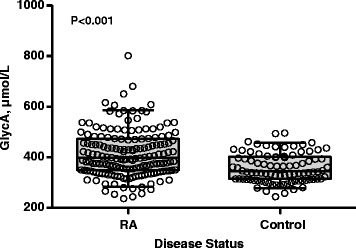
GlycA is higher in patients with rheumatoid arthritis compared with control subjects. Boxes represent the median (middle horizontal line) and the 25th and 75th percentiles. Whiskers represent the 5th and 95th percentiles. Each data point is presented as an open circle. Patients with rheumatoid arthritis had a higher GlycA concentration (median (interquartile range): 398 μmol/L (348 to 473 μmol/L)) compared with control subjects (344 μmol/L (314 to 403 μmol/L); *P* < 0.001)

Patients with RA and control subjects were of similar age, race and sex (Table 1). In patients with RA, the median (IQR) DAS28-ESR and DAS28-CRP were 3.86 units (2.63 to 4.90 units) and 3.09 units (2.06 to 3.80 units), respectively. Patients with RA had higher CRP concentrations than control subjects (*P* < 0.001). The majority of patients with RA were taking methotrexate (71 %) and prednisone (54 %), and 20 % were taking an anti-TNFα agent (Table 1). Approximately 10 % of both RA and control subjects had known coronary artery disease, and coronary artery calcium was present in 52 % of patients with RA and 39 % of control subjects. GlycA concentrations were higher in patients with RA (median (IQR): 398 μmol/L (348 to 473 μmol/L)) compared with control subjects (344 μmol/L (314 to 403 μmol/L)) (*P* < 0.001) (Fig. 1).

**Table 1:** Participant characteristics^a^

	RA (n = 166)	Control (n = 90)	*P*-value
Median age (IQR), yr	54 (45 to 63)	53 (44 to 60)	0.38
Caucasian race, n (%)	147 (89)	77 (86)	0.55
Female sex, n (%)	114 (69)	56 (62)	0.30
RF-positive, n (%)	114 (72)	–	–
Median DAS28-ESR (IQR)	3.86 (2.63–4.90)	–	–
Median DAS28-CRP (IQR)	3.09 (2.06–3.80)	–	–
Tender joints, n (IQR)	2 (0 to 7)	–	–
Swollen joints, n (IQR)	3 (0 to 8)	–	–
Median CRP (IQR), mg/L	4.0 (1.2 to 11)	0.5 (0.2 to 1.5)	<0.001
Median ESR (IQR), mm/hr	15 (7 to 36)	–	–
Known CAD, n (%)	17 (10)	9 (10)	0.95
Coronary artery calcium, n (%)	83 (52)	34 (39)	0.05
Coronary calcium score, Agatston units	2.7 (0 to 178.7)	0 (0 to 19.2)	0.02
Methotrexate use, n (%)	118 (71)	–	–
Leflunomide use, n (%)	29 (18)	–	–
Hydroxychloroquine use, n (%)	42 (25)	–	–
Anti-TNFα agent use, n (%)	33 (20)	–	–
Prednisone use, n (%)	89 (54)	–	–
NSAID use, n (%)	55 (33)	33 (37)	0.53

^a^RF data were available in 159 patients with RA. Coronary artery calcium scores were available for 161 patients with RA and 88 control subjects. CAD, Coronary artery disease; CRP, High-sensitivity C-reactive protein; DAS28, Disease Activity Score based on 28 joints; ESR, Erythrocyte sedimentation rate; IQR, Interquartile range; NSAID, Non-steroidal anti-inflammatory drug; RA, Rheumatoid arthritis; RF, Rheumatoid factor; TNFα, Tumor necrosis factor α

### GlycA relationship to disease activity in rheumatoid arthritis and disease-related factors

**Fig. 2:**
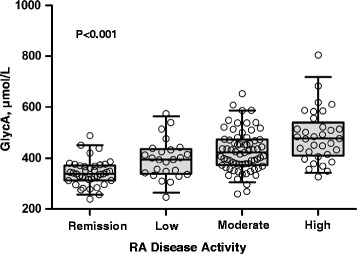
GlycA increases with increasing rheumatoid arthritis disease activity. Boxes represent the median (middle horizontal line) and the 25th and 75th percentiles. Whiskers represent the 5th and 95th percentiles. Each data point is presented as an open circle. Rheumatoid arthritis (RA) disease activity on the basis of Disease Activity Score based on 28 joints using erythrocyte sedimentation rate (DAS28-ESR) was defined as follows: remission = DAS28 < 2.6 (n = 39); low = DAS28 between 2.6 and 3.2 (n = 25); moderate = DAS28 between 3.2 and 5.1 (n = 67); and high = DAS28 > 5.1 (n = 33)

Increasing GlycA concentrations were significantly associated with increasing RA disease activity (*P* < 0.001) (Fig. 2). GlycA was correlated with DAS28-ESR (Spearman ρ = 0.58, *P* < 0.001) and DAS28-CRP (ρ = 0.47, *P* < 0.001). These correlations were similar or slightly stronger than those between CRP and DAS28-ESR (ρ = 0.48, *P* < 0.001) and between ESR and DAS28-CRP (ρ = 0.36, *P* < 0.001) (Table 2). Similarly, the cross-sectional relationship between Larsen score and GlycA (ρ = 0.24, *P* = 0.02) was similar or slightly stronger than the relationship with CRP (ρ = 0.18, *P* = 0.09) or ESR (ρ = 0.16, *P* = 0.13) (Table 2).

**Table 2:** Association between GlycA, C-reactive protein and erythrocyte sedimentation rate with measures of disease activity and clinical characteristics in patients with rheumatoid arthritis^a^

	GlycA	ESR	CRP
	ρ	ρ	ρ
DAS28-ESR	0.58^b^	0.65^b^	0.48^b^
DAS28-CRP	0.47^b^	0.36^b^	0.50^b^
Tender joints	0.28^b^	0.12	0.16^c^
Swollen joints	0.29^b^	0.28^b^	0.29^b^
Global health score	0.25^b^	0.21^c^	0.22^c^
Larsen score^d^	0.24^c^	0.16	0.18
ESR	0.64^b^	–	0.59^b^
CRP	0.61^b^	0.59^b^	–
IL-6	0.39^b^	0.38^b^	0.40^b^
TNFα	0.25^c^	0.28^b^	0.21^c^
Age	0.04	0.18^c^	0.08
BMI	0.08	0.09	0.17^c^
Hemoglobin	−0.12	−0.51^b^	−0.21^c^
eGFR	0.02	−0.04	0.05

^a^Correlation data are based on Spearman’s correlation coefficient (ρ). BMI, Body mass index; CRP, High-sensitivity C-reactive protein; DAS28, Disease Activity Score based on 28 joints; eGFR, Estimated glomerular filtration rate; ESR, Erythrocyte sedimentation rate; IL-6, Interleukin-6; TNFα, Tumor necrosis factor α. ^b^ *P* ≤ 0.001. ^c^ *P* < 0.05. ^d^Larsen scores were available for 92 patients with RA

The AUC for GlycA’s ability to discriminate between patients with low versus moderate to high disease activity was 0.78 (95 % CI: 0.71, 0.86) based on DAS28-ESR and 0.75 (0.68, 0.83) based on DAS28-CRP. Such discrimination was similar or better with GlycA compared with ESR (AUC: 0.68 (95 % CI: 0.60, 0.76)) using DAS28-CRP and compared with CRP (AUC: 0.74 (95 % CI: 0.67, 0.82)) using DAS28-ESR. GlycA was also associated with components of the DAS28 scores including tender and swollen joint counts, patient-reported global health score, ESR and CRP (all *P* < 0.001) (Table 2).

The relationship between GlycA concentrations and radiographic damage was assessed in a subset of 92 patients with RA for whom radiographic data were obtained. GlycA was significantly correlated with Larsen score (ρ = 0.24, *P* = 0.02) (Table 2). GlycA concentration was not significantly different between rheumatoid factor–positive (median (IQR) 408 μmol/L (351 to 479 μmol/L) and rheumatoid factor–negative patients with RA (385 μmol/L (336 to 452 μmol/L) (*P* = 0.09). Also, there were no significant differences in GlycA concentration between patients with RA who were receiving methotrexate, leflunomide, hydroxychloroquine, corticosteroids, anti-TNFα agents, non-steroidal anti-inflammatories, anti-hypertensives or statins compared with those not receiving the respective drugs (all *P* > 0.07).

### Relationship between GlycA and subclinical atherosclerosis in rheumatoid arthritis

We examined the cross-sectional relationship between GlycA and the presence of coronary artery calcium, a marker of subclinical atherosclerosis (Table 3). Among patients with coronary artery calcium, the median (IQR) GlycA concentration was 415 μmol/L (356 to 477 μmol/L), which trended higher than the median GlycA concentration of those without coronary calcium (379 μmol/L ([337 to 457 μmol/L)) (*P* = 0.06). For each quartile increase in GlycA, the odds of having coronary artery calcium increased by 48 % (95 % CI: 4 %, 111 %), independent of age, race and sex (*P* = 0.03). This relationship was attenuated after adjustment for traditional CV risk factors, including age, sex, total cholesterol, HDL cholesterol, smoking status, systolic blood pressure and anti-hypertensive use (*P* = 0.07), and additionally for diabetes (*P* = 0.12). Although only 17 of 166 patients with RA had a diagnosis of established coronary artery disease, we performed an exploratory analysis to determine the relationship between GlycA and prevalent coronary artery disease. Among patients with coronary artery disease, the median (IQR) GlycA concentration was 453 μmol/L (380 to 487 μmol/L), which was significantly higher than the median GlycA concentration of those without coronary artery disease (395 μmol/L (344 to 487 μmol/L) (*P* = 0.03). Similarly, with each quartile increase in GlycA, the odds of having a diagnosis of coronary artery disease increased 110 % (17 % to 277 %), independent of age, race and sex (*P* = 0.01), and remained significant with additional adjustment for traditional CV risk factors (*P* = 0.01) and additionally for diabetes (*P* = 0.02) (Table 3).

**Table 3:** Relationship between GlycA and cardiovascular disease^a^

GlycA	OR (95 % CI)	*P*-value	OR^b^ (95 % CI)	Adjusted *P*-value^b^	OR^c^ (95 % CI)	Adjusted *P*-value^c^	OR^d^ (95 % CI)	Adjusted *P*-value^d^
Coronary calcium	1.32 (0.99, 1.74)	0.06	1.48 (1.04, 2.11)	0.03	1.39 (0.97, 2.01)	0.07	1.35 (0.93, 1.95)	0.12
Known CAD	1.83 (1.10, 3.03)	0.02	2.10 (1.17, 3.77)	0.01	2.14 (1.17, 3.92)	0.01	2.10 (1.13, 3.88)	0.02

^a^All analyses were performed using logistic regression. CAD, Coronary artery disease; CI, Confidence interval; OR, Odds ratio per quartile increase in GlycA concentration. ^b^Adjusted for age, race and sex. ^c^Adjusted for age, sex, total cholesterol, high-density lipoprotein (HDL) cholesterol, smoking status, systolic blood pressure and anti-hypertensive use. ^d^Adjusted for age, sex, total cholesterol, HDL cholesterol, smoking status, systolic blood pressure, anti-hypertensive use and diabetes

### Relationship between potential confounders and GlycA

As discussed above, CRP and ESR have been reported to be altered by age, sex and BMI. We found no significant association between either age or BMI and GlycA concentration (all *P* ≥ 0.30) (Table 2), and we observed no difference between men and women (*P* = 0.31). Similarly, because anemia and impaired renal function may alter ESR, we compared the association between GlycA and hemoglobin and eGFR. There was no significant correlation between GlycA and hemoglobin or eGFR (all *P* > 0.60) (Table 2). Conversely, CRP was significantly correlated with BMI (positively) and hemoglobin (inversely), and ESR was significantly correlated with age (positively) and hemoglobin (inversely) (Table 2). Neither CRP nor ESR was significantly correlated with eGFR. However, most patients in this cohort had normal renal function (median eGFR: 87 ml/min/1.73 m^2^ (IQR: 74 to 103 ml/min/1.73 m^2^), range: 28 to 161 ml/min/1.73 m^2^).

## Discussion

The major new findings of the present study are that GlycA may be a useful marker of disease activity and CV risk in patients with RA. GlycA was strongly correlated with all components of DAS28 scores and was correlated with radiographic damage in cross-sectional analysis. Moreover, it was associated with the presence of coronary artery calcium and prevalent coronary artery disease in patients with RA.

GlycA is measured from an NMR signal that primarily represents glycosylated acute-phase proteins [10, 18]. Glycosylation, not to be confused with the non-enzymatic binding of simple sugars as occurs with hemoglobin A1c in patients with diabetes mellitus [40], is an enzymatic modification whereby an oligo or polysaccharide moiety is attached to a protein or lipid. This modification has an important role in protein folding and stabilization, cell signaling, cellular adhesion and antigen recognition [41]. In fact, during an acute-phase response, not only are levels of serum acute phase glycoproteins altered, but their glycan structures are also dynamically modified by circulating glycosidases and glycosyltransferases [42, 43]. Therefore, the measurement of these protein glycans via NMR (GlycA) incorporates alterations in both protein and glycan concentrations that occur during inflammatory responses.

Previous studies have identified glycosylation products as markers of RA and inflammatory disease. In one study [19], 47 patients with RA and 51 control subjects were followed longitudinally and metabolic profiles using NMR spectroscopy were assessed over time from plasma samples. Peaks arising from glycosylated proteins (including the GlycA signal), cholesterol, lactate and unsaturated lipids appeared to be biomarkers of the presence of RA, but these measures used together were not robust markers of changes in RA disease activity [19]. Others found that NMR measurements of total glycosylated residues (including the signal for GlycA) and of *N*-acetylglucosamine (a component of GlycA) plus *N*-acetylneuraminic acid were elevated in 21 patients with rheumatic or infectious diseases compared with 186 control subjects or patients with cancer [20]. Although previous authors have shown interesting correlations between the NMR signal arising from glycosylated proteins and various disease states, the standardized measurement of GlycA from the profile spectra obtained on a clinical NMR analyzer allows for more robust clinical investigations.

Given that the GlycA peak represents glycosylated acute-phase proteins, it is not surprising that GlycA is closely associated with disease activity in RA. Interestingly, GlycA correlated at least as well as CRP or ESR with several measures of disease activity, radiographic damage and presence of coronary calcium. Moreover, GlycA had similar discriminative capacity to differentiate between low and moderate to high disease activity (AUC: 0.75) compared with published data for the multibiomarker disease activity test (AUC: 0.76) in which CRP was used as a component of both the predictor multibiomarker test and outcome of disease activity measured by DAS28-CRP [44].

Correlations with disease activity in a cross-sectional study may be stronger with GlycA than with CRP or ESR if GlycA is a more stable marker of inflammation that is less susceptible to alterations by both day-to-day variability and non-inflammatory factors. Indeed, the day-to-day variability of GlycA concentrations is lower than for CRP [10]. The speed at which GlycA rises and falls after an inflammatory stimulus is not known; however, given its components, it is likely that GlycA rises over several days and falls over several weeks. This is similar to the pattern of ESR’s rise and fall due to fibrinogen’s impact on ESR [45], though fibrinogen is not a component of GlycA. Interestingly, GlycA appears to be influenced less by non-inflammatory conditions such as age, anemia, and BMI than by CRP or ESR.

Our findings in patients with RA are concordant with findings in the general population. GlycA was measured in participants of the Women’s Health Study, which included 27,491 women with a median of 17.2 years of follow-up [21]. GlycA concentrations were correlated strongly with CRP (ρ = 0.61, *P* < 0.0001), and baseline GlycA concentrations were associated with incident CV events. For example, within the first 6 years of follow-up, the hazard ratio Q4 for CV events was 1.34 (95 % CI: 1.22, 1.48) (P < 0.0001 for each 1 SD increase in GlycA). Notably, this unit of increase is similar to the observed difference in GlycA concentration between RA and control subjects in the present study.

We have evaluated the use of a novel inflammatory marker for utility in assessing disease activity and CV risk. There are other novel biomarkers that have similarly been proposed as important biomarkers of both RA disease activity and atherosclerosis or CV disease. Some recent examples include osteoprotegerin [46, 47] and angiopoietin-2 [48].

Although we included a relatively large cohort of patients with RA who were well characterized with respect to inflammation and CV risk, the present study has limitations. The adjusted analyses for the relationship between GlycA and prevalent coronary artery disease should be interpreted with caution because models were overfitted, owing to the relatively small number of patients with RA and prevalent coronary artery disease (N = 17). Because this was a cross-sectional study, the longitudinal implications of the findings regarding an association are uncertain. For example, although the finding that GlycA was associated with radiographic damage is interesting, additional studies are required to determine if GlycA has utility as a marker of the risk of radiographic progression. Moreover, future investigation of GlycA’s ability to measure changes in disease activity in response to therapy and development of CV events is important to fully evaluate its utility in patients with RA.

## Conclusions

GlycA is increased in patients with RA compared with control subjects. It is strongly correlated with DAS28 and its components, and it is also correlated with radiographic damage. Moreover, increased GlycA concentrations are associated with the presence of coronary artery calcification.

## Abbreviations

AUC: Area under the curve
BMI: Body mass index
CAD: Coronary artery disease
CI: Confidence interval
CRP: C-reactive protein
CV: Cardiovascular
DAS28: Disease Activity Score based on 28 joints
eGFR: Estimated glomerular filtration rate
ELISA: Enzyme-linked immunosorbent assay
ESR: Erythrocyte sedimentation rate
HDL: High-density lipoprotein
IL-6: Interleukin-6
IQR: Interquartile range
NMR: Nuclear magnetic resonance
NSAID: Non-steroidal anti-inflammatory drug
OR: Odds ratio
RA: Rheumatoid arthritis
RF: Rheumatoid factor
SD: Standard deviation
TNFα: Tumor necrosis factor α
